# High-Level Expression of a Thermally Stable Alginate Lyase Using *Pichia pastoris*, Characterization and Application in Producing Brown Alginate Oligosaccharide

**DOI:** 10.3390/md16050158

**Published:** 2018-05-11

**Authors:** Haifeng Li, Shuling Wang, Yunyi Zhang, Liehuan Chen

**Affiliations:** 1 College of Medicine, Hangzhou Normal University, Hangzhou 311121, China; lihf@hznu.edu.cn (H.L.); wsling222@163.com (S.W.); 2 Department of Microbiology, Zhejiang Provincial Center for Disease Control and Prevention, Hangzhou 310051, China; 3 College of Animal Sciences and Technology, Zhongkai University of Agriculture and Engineering, Guangzhou 510225, China

**Keywords:** *Pichia pastoris*, alginate lyase, expression, oligosaccharide

## Abstract

An alginate lyase encoding gene *sagl* from *Flavobacterium* sp. H63 was codon optimized and recombinantly expressed at high level in *P.pastoris* through high cell-density fermentation. The highest yield of recombinant enzyme of *sagl* (rSAGL) in yeast culture supernatant reached 226.4 μg/mL (915.5 U/mL). This was the highest yield record of recombinant expression of alginate lyase so far. The rSAGL was confirmed as a partially glycosylated protein through EndoH digestion. The optimal reaction temperature and pH of this enzyme were 45 °C and 7.5; 80 mM K^+^ ions could improve the catalytic activity of the enzyme by 244% at most. rSAGL was a thermal stable enzyme with T50^15^ of 57–58 °C and T50^30^ of 53–54 °C. Its thermal stability was better than any known alginate lyase. In 100 mM phosphate buffer of pH 6.0, rSAGL could retain 98.8% of the initial activity after incubation at 50 °C for 2 h. Furthermore, it could retain 61.6% of the initial activity after 48 h. The specific activity of the purified rSAGL produced by *P. pastoris* attained 4044 U/mg protein, which was the second highest record of alginate lyase so far. When the crude enzyme of the rSAGL was directly used in transformation of sodium alginate with 40 g/L, 97.2% of the substrate was transformed to di, tri, tetra brown alginate oligosaccharide after 32 h of incubation at 50 °C, and the final concentration of reducing sugar in mixture reached 9.51 g/L. This is the first report of high-level expression of thermally stable alginate lyase using *P. pastoris* system.

## 1. Introduction

Alginate is a kind of linear anionic polymer composed of α-l-guluronate and its C5 epimer β-d-mannuronate. Alginate is the major cell wall polysaccharides of brown algae. Alginate lyase can catalyze the degradation of alginate to produce oligosaccharides, which have been confirmed to have many biological activities [1]. Brown alginate oligosaccharide has been applied extensively in food, medicine, and agriculture. Alginate lyases with high catalytic activity are the key factors during the process of oligosaccharides production using alginate as raw materials. Meanwhile, sufficient supply of alginate lyase is also important for oligosaccharides production. Alginate lyases have been found in many marine bacteria, fungi, and invertebrate. A variety of types of wild microorganisms which could produce alginate lyase had been found and investigated. However, low yield of alginate lyases in these isolates limited their applications. Recombinant expression of alginate lyases in engineering microorganisms is a good resolution for this problem. To date, some alginate lyases have been successfully recombinantly expressed in *Escherichia coli* system [1,2]. However, no high enzyme yields satisfying industrial need were obtained in these reports. Furthermore, the recombinant protein produced by *E. coli* usually was not be recommended to use in food and feed industry because of safety of residual endotoxin. Comparing to *E. coli*, *Pichia pastoris* was used to produce enzyme for pharmaceutical and food industries more frequently in the past two decades. This system had advantages of efficient extracellular protein secretion capacity, convenient product separation process, and no endotoxin production. In one report, a chitosanase from *Bacillus subtilis* HD145 was recombinantly produced by *Pichia pastoris* system. The yield reached 800 mg/L after optimization of fermentation conditions [3]. *Pichia pastoris* system usually is first choice in production of industrial-scale recombinant proteins, but there were almost no reports of alginate lyases recombinantly expressed in *P. pastoris* system so far. Exploring the potential application of *P. pastoris* in alginate lyase production is worth studying. In this study, an alginate lyase gene *sagl* from *Flavobacterium* sp. H63 was successfully expressed in *P. pastoris* with high-level yield. Before now, this *sagl* gene has not been well expressed in *E. coli* system because of an inclusion body problem. Meanwhile, the characteristics of this recombinant alginate lyase produced by yeast were investigated in detail. In addition, the large-scale production of brown alginate oligosaccharide using this recombinant enzyme was studied.

## 2. Results and Discussion

### 2.1. Recombinant Expression of SAGL in Escherichia coli

**Figure 1:**
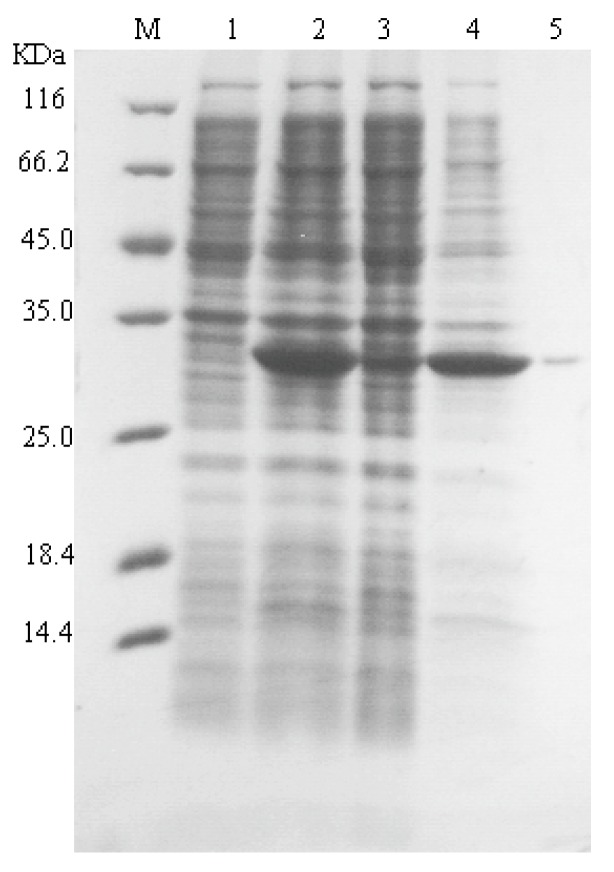
SDS-PAGE analysis of SAGL expression in the *E. coli* system. Lane M protein marker; lane 1 cell lysate of BL21(DE3); lane 2 whole cell lysate of BL21(DE3) with pET28a-vectors; lane 3 supernatant of cell lysate with recombinant vectors; lane 4 precipitate of cell lysate with recombinant vectors; lane 5 purified recombinant alginate lyase.

Recombinant *E. coli* carrying pET28a-nagl vector was induced and tested by SDS-PAGE. Most recombinant alginate lyase were in insoluble inclusion body form in *E. coli* of BL21(DE3) (Figure 1). This recombinant alginate lyase in supernatant of cell lyaste was purified using Ni-NTA resin (Figure 1, lane 5). The yield of only 10 units per mL culture was obtained using *E. coli* system.

### 2.2. Recombinant Expression of SAGL Using P. pastoris in Flask

**Figure 2:**
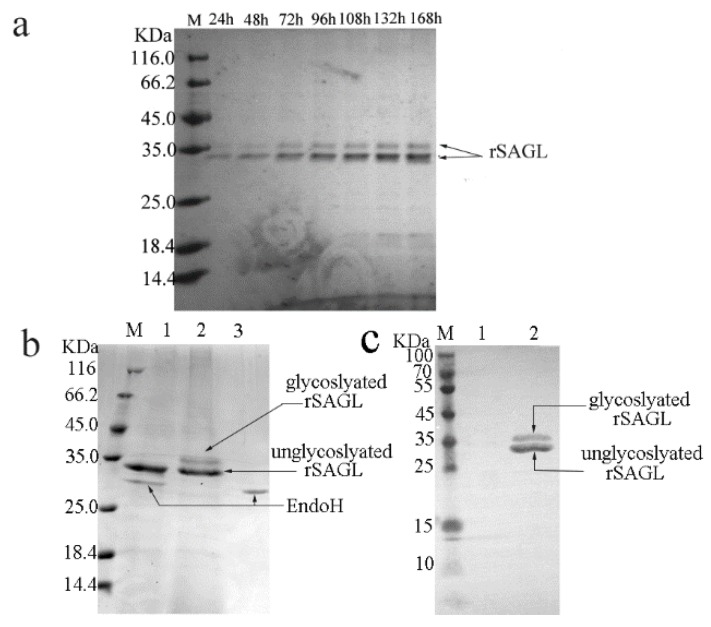
Expression in flask fermentation, glycosylation, and western-blotting analysis of rSAGL. (**a**) SDS-PAGE analysis of SAGL expression in shaking flask. BMMY medium was used in shaking flask. Culture supernatant samples collected at 24 h intervals were tested by electrophoresis. The target protein bands represented rSAGL were marked by arrows; (**b**) glycosylation analysis of purified rSAGL. Lane M protein marker; lane 1 unglycosylated rSAGL, EndoH protein band were marked by arrows; lane 2 purified rSAGL control, glycosylated and unglycosylated rSAGL protein band were marked by arrows; lane 3 EndoH blank control; (**c**) western -blotting of purified rSAGL. Lane M protein marker; lane 1 BSA control; lane 2 purified rSAGL.

A linearized recombinant vector pPICZαA carrying *sagl* alginate lyase gene was transformed into *P. pastoris* X33 competent cells by using electroporation. More than 400 positive yeast colonies selected using PCR were inoculated on screening plates. A yeast colony with the largest hydrolytic circle around the colony was selected. The ratio of the diameter of hydrolytic circle to that of this colony reached about 4:1. The shaking flask fermentation of this strain proceeded. After 168 h of fermentation, the activity of recombinant SAGL protein in supernatant reached maximum of 93.5 U/mL. The protein expression of this strains in shaking flask was analyzed by using SDS-PAGE. Two protein bands with molecular weight (MW) of 35 KDa and 32 KDa were detected in crude supernatant samples from recombinant strain without concentration treatment (Figure 2a), but the protein content of the 35 KDa band was much lower than that of the 32 KDa band. After purification using Ni-NTA resin, the enzyme was still constituted of two bands of 35 KDa and 32 KDa (Figure 2b). The value of 32 KDa was consistent with calculated MW of recombinant SAGL alginate lyase, whereas after deglycosylation treatment using Endo H two protein bands comigrated into one 32 KDa band in SDS-PAGE (Figure 2b). Next, mouse anti His antibody was used in Western blotting. Two blots represented two protein bands of recombinant SAGL were developed (Figure 2c). It was confirmed that two protein bands both contained histidine tag. The 32 KDa band was unglycosylated recombinant SAGL protein and the 35 KDa band consisted of glycosylated ones.

Glycosylation of recombinant proteins expressed in *Pichia* yeast has been reported previously. For example, a chitosanase from *Bacillus subtilis* HD145 was recombinantly expressed in *P. pastoris*. The recombinant chitosanase enzyme was also partially glycosylated and constituted of two protein bands with 33 KDa and 31 KDa. [3]. It was confirmed that thermal stability of recombinant chitosanase was better than native enzyme from *Bacillus subtilis* extract. The author speculated that the improvement of thermal stability was caused by the glycosylation of protein. These results in Figure 2 showed that SAGL alginate lyase was successfully expressed in *P. pastoris* in flask. At the end of fermentation, the yield of alginate lyase in flask reached 23.1 μg/mL culture (about 95 U/mL at optimum testing condition). There was only one public report about the recombinant expression of alginate lyase using *P. pastoris* system [4]. The alginate lyase gene paAlgL from *Pseudomonas aeruginosa* PAOI was recombinantly expressed in *P. pastoris* GS115, and the specific activity of the recombinant paAlgL reached 2440 U/mg protein, but no yield data was shown. In that paper the reason of choosing *P. pastoris* rather than *E. coli* was that the recombinant target proteins in the latter system tended to be inclusion body and the complicated purification procedures caused significant decrease in yield of recombinant alginate lyase. The recombinant paAlgL alginate lyase produced by *Pichia* yeast was not glycosylated and there were no comparison results between recombinant alginate lyase produced by *P. pastoris* and *E. coli* in that paper.

### 2.3. Characterization of Recombinant sagl from E. coli and P. pastoris Systems

**Figure 3:**
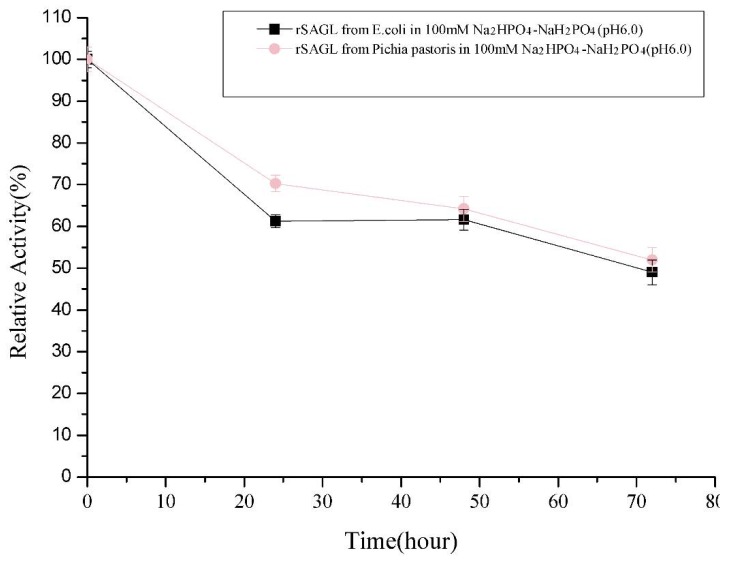
Comparison of thermal stability at 50 °C between rSAGL from *E. coli* and rSAGL from *P. pastoris*. The purified recombinant enzymes from above two microorganisms were stored in 100 mM Na_2_HPO_4_-NaH_2_PO_4_ buffer (pH 6.0) and incubated at 50 °C for 72 h. All experiments were conducted in triplicate, and the data were expressed as mean ± standard deviation.

In our early flask experiment, the expression of *sagl* gene in *E. coli* BL21(DE3) was not well, although we made a lot of efforts including changing of expression vector, host strain, and gene codons optimization (data not shown). The main target enzyme was still expressed as inclusion body form (Figure 1, line 4). After Ni-affinity chromatography purification, the yield of recombinant alginate lyase was determined as about 10 units per microliter culture. This yield was too low to apply the recombinant enzyme in scale oligosaccharide production, so we chose the *P. pastoris* system and obtained significant improvement of yield. For detailed characterization of rSAGL expressed by yeast, rSAGL produced by *E. coli* was purified and used as a comparison of non-glycosylated rSAGL. After determination, the specific activity of partially glycosylated rSAGL from *P. pastoris* and non-glycosylated rSAGL from *E. coli* were almost equal (2080 U/mg protein vs. 2133 U/mg protein). The *K*m values against sodium alginate of the two enzymes were almost same (4.63 mg/mL vs. 4.64 mg/mL). Concerning thermal stability, there was no significant difference between the two enzymes when they were incubated at 50 °C in 100 mM Na_2_HPO_4_-NaH_2_PO_4_ buffer (pH 6.0). They could both maintain about 60% and 50% of initial activity at 48 h and 72 h (Figure 3). It was concluded that partial glycosylation of rSAGL had no effect on its thermal stability, specific activity, and substrate affinity ability against sodium alginate. rSAGL produced by *P. pastoris* and *E. coli* both were thermally stable at 50 °C. Partial glycosylation had no obvious impact on the stability of rSAGL produced by *P. pastoris*.

**Figure 4:**
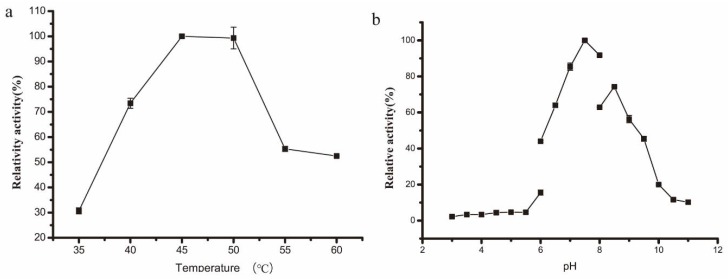
Optimization of catalytic temperature (**a**) and pH (**b**) of rSAGL from *P. pastoris.* Na_2_HPO_4_-NaH_2_PO_4_ buffer (100 mM, pH 6.0) and sodium alginate were used as standard buffer and standard substrate in tests. All experiments were conducted in triplicate, and the data were expressed as mean ± standard deviation.

The optimal reaction temperature of this rSAGL enzyme was determined as 45 °C (Figure 4a). Most known alginate lyases were not thermally stable; they exhibited optimal catalytic activity between 30 °C and 40 °C. There were few thermophilic alginate lyase. A recombinant alginate lyase from *Saccharophagus degradans* had optimum catalytic temperature of 50 °C, but it lost 58% of initial activity at 50 °C after 30 min [5]. A recombinant alginate lyase from *Nitratiruptor* sp. SB155-2 possessed optimum catalytic temperature of 70 °C, but it lost 80% of initial activity by incubation at 50 °C for 16 h [6]. The optimal catalytic pH of rSAGL was 7.5 (Figure 4b). Most known alginate lyases exhibited best catalytic activity between pH 6.0 and 8.0.

Metal ions can affect enzyme activity by large extent. In our test, most metal ions could inhibit enzyme activity of rSAGL, except for Mg^2+^, Na^+^, and K^+^. These three kinds of ions all could improve activity by different degrees. Mg^2+^ of 100 mM could improve the catalytic activity of rSAGL by 47% (Table 1).

Na^+^ and K^+^ ions known as activator of some alginate lyases could significantly improve the activity of rSAGL. K^+^ ions of 80 mM were found to have the best catalytic activity improvement of rSAGL by 244% (Table 2). By combination of the above parameters, the specific activity of rSAGL was improved from 2080 U/mg to 4044 U/mg under optimum catalytic conditions including 45 °C, 80 mM K_2_HPO_4_-KH_2_PO_4_ buffer (pH 7.5) and 10 g/L sodium alginate substrate. This specific activity was the second highest record of alginate lyase activity by comparing with known alginate lyases [7].

### 2.4. End Product Analysis

**Figure 5:**
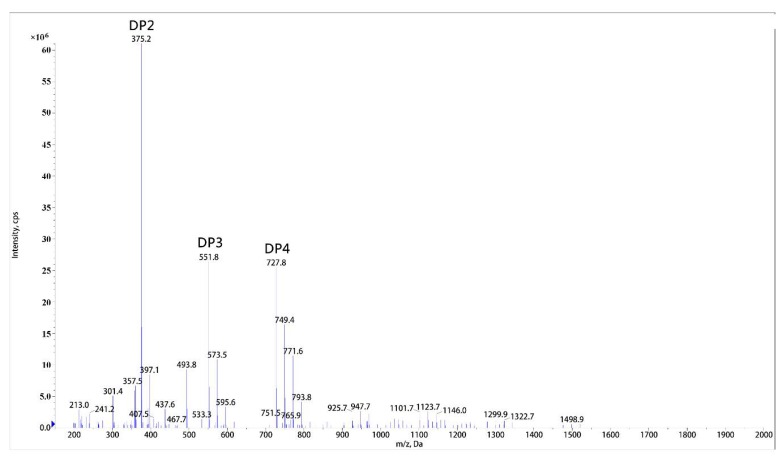
ESI-MS analysis of end hydrolysis product of rSAGL. Sodium alginate was used as substrate in test. DP2,3,4 peak represented disaccharide, trisaccharide, tetrasaccharide of alginate oligosaccharide. DP2 (disaccharide *m*/*z* 352 + Na^+^: *m*/*z* 375.2), DP3 (trisaccharide *m*/*z* 528 + Na^+^: *m*/*z* 551.8), and DP4 (tetrasaccharide *m*/*z* 704 + Na^+^: *m*/*z* 727.8).

Through ESI-MS analysis of molecular weight, the end hydrolysis products of sodium alginate substrate were determined as mixture of disaccharide, trisaccharide, and tetrasaccharide (Figure 5). These oligosaccharides existed as sodium salt form. The catalytic mode of rSAGL was confirmed as endo manner because no monosaccharide product was found. The product spectrum of rSAGL was similar to that of most endo-type alginate lyase. The spectrums of these enzymes were all focused on a range of 2–7 polymerization degree with low molecular weight, and it was acknowledged that oligosaccharide with low molecular weight usually had better biological activity.

### 2.5. Characterization of Thermal Stability of rSAGL

**Figure 6:**
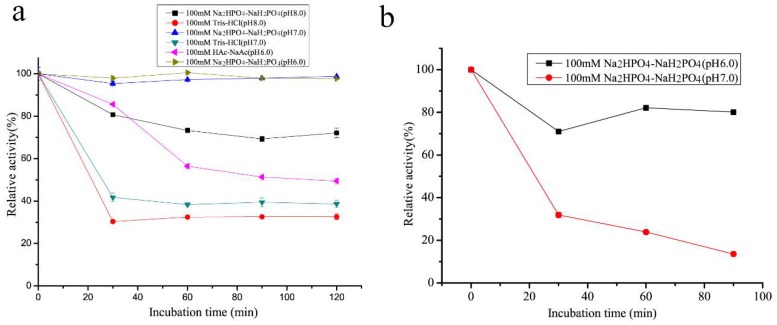
Effect comparison of different buffers on thermal stability of rSAGL from *P. pastoris*. Two temperatures of 50 °C (**a**) and 55 °C (**b**) were used in the test. In the residual activity test, Na_2_HPO_4_-NaH_2_PO_4_ buffer (100 mM, pH 6.0) and sodium alginate (10 g/L) were used at 50 °C. All experiments were conducted in triplicate, and the data were expressed as mean ± standard deviation.

The thermal stability of rSAGL from *P. pastoris* was impressive. It could retain 49.0% of initial activity after incubation at 50 °C in phosphate salt buffer (pH 6.0) for 72 h (Figure 3). In the same buffer, T50 values of rSAGL at 15 min and 30 min were determined as 57–58 °C (T50^15^) and 53–54 °C (T50^30^). Next, it was necessary to verify if the incubation buffer had significant effects on the thermal stability of rSAGL. The result in Figure 6a indicated that 100 mM Na_2_HPO_4_-NaH_2_PO_4_ buffer (pH 6.0 and pH 7.0) had better performance than Tris-HCl and HAc-NaAc buffer in the thermal stability test at 50 °C for 120 min. When the incubation temperature was increased to 55 °C, rSAGL also could retain 78.9% of initial activity after 1.5 h incubation in phosphate salt buffer of pH 6.0 (Figure 6b). However, the enzyme sample in phosphate salt buffer of pH 7.0 only retain 12.1% of initial activity after 1.5 h incubation at 50 °C. It was concluded that phosphate salt buffer with pH 6.0 was most advantageous to the stability of rSAGL. This enzyme was very stable in this buffer at temperatures lower than 50 °C.

During the process of alginate lyases catalysis, high catalytic temperature can facilitate the transformation of substrate due to the reduction of viscosity of reaction mixture and improvement of enzyme activity. High process temperature (above 45 °C) can also prevent microorganism contamination when crude substrate such as kelp powder is transformed. Alginate lyases with good thermal stability are very valuable for enzymatic production of brown algae oligosaccharide. However, most known alginate lyases are not thermally stable. The possible reason was that they were mainly from marine bacteria which were isolated from low temperature locations [7,8,9,10,11].

### 2.6. Comparison of Catalytic Property

The specific activity and thermal stability of rSAGL were impressive. It was necessary to compare its catalytic property with some reported endo-type alginate lyases [1,2,4,5,6,7,8,9,10,11,12,13,14,15,16]. The property parameters of these alginate lyase enzymes are shown in Table 3. The specific activity of rSAGL (4044 U/mg) was the second highest record among known alginate lyases. The thermal stability of rSAGL produced by *P. pastoris* at 50 °C was also the highest comparing other enzymes in Table 3. These catalytic properties made rSAGL a promising enzyme for the transformation of sodium alginate to produce brown alginate oligosaccharide.

### 2.7. Phylogenetic Tree Analysis of Sagl Based on Amino Acid Sequence

**Figure 7:**
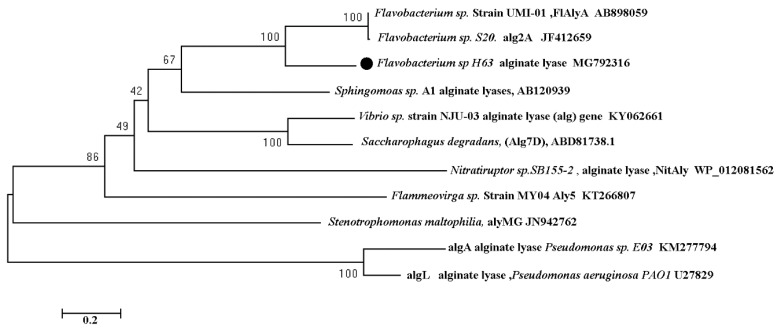
Phylogenetic tree analysis of *sagl* based on amino acid sequence. The neighbor-joining method was used in construction.

In order to analyze the evolution relationship of *sagl* (MG792316) with known alginate lyases, a phylogenetic tree was constructed (Figure 7). Some alginate lyases in Table 3 were selected in construction. *sagl* (MG792316) has the nearest relationship with two alginate lyases AB898059 and JF412659, which were also from *Flavobacterium* genus. Alginate lyase of *Pseudomonas aeruginosa* PAOI (U27829), which was the first alginate lyase recombinantly expressed using *P. pastoris* system, had the farthest evolutionary relationship with *sagl*. The thermal stability difference between two recombinant alginate lyases perhaps were results of far evolutionary distance.

### 2.8. High Density Fermentation

**Figure 8:**
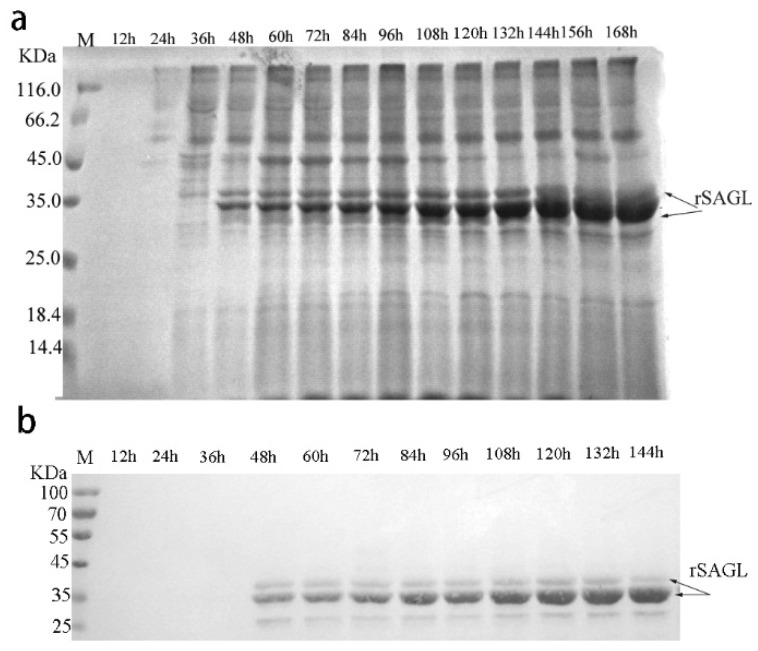
Expression analysis of recombinant SAGL in fermentor. (**a**) SDS-PAGE analysis of SAGL expression in fermentor. BSM medium was used in test. Culture supernatant samples collected at 12 h intervals were tested by electrophoresis. The target protein bands represented rSAGL were marked by arrows; (**b**) Western blot of culture supernatant of recombinant yeast. Lane M protein marker; culture samples from fermenters at 12, 24, 36, 48, 60, 72, 84, 96, 108, 120, 132, and 144 h were tested in turn at the following lanes.

In previous reports, the yields of some recombinant proteins produced by *Pichia* yeast reached gram level through various parameters optimization. In order to obtain higher yield of recombinant alginate lyase of *sagl*, fed-batch fermentation was carried out in 10 L fermentor according to standard high-density fermentation protocol. Firstly, the culture samples were collected at 12 h interval and the protein expression in fermentation was tested by SDS-PAGE and western-blotting. The results are shown in Figure 8a. From 48 h of fermentation, recombinant SAGL including glycosylated and unglycosylated bands both could be detected. The amount of unglycosylated rSAGL was more and more until end of 168 h fermentation. However, after 144 h, the glycosylated band was gradually not detected. Western blot results in Figure 8b confirmed again that 35 KDa and 32 KDa bands both are rSAGL protein.

**Figure 9:**
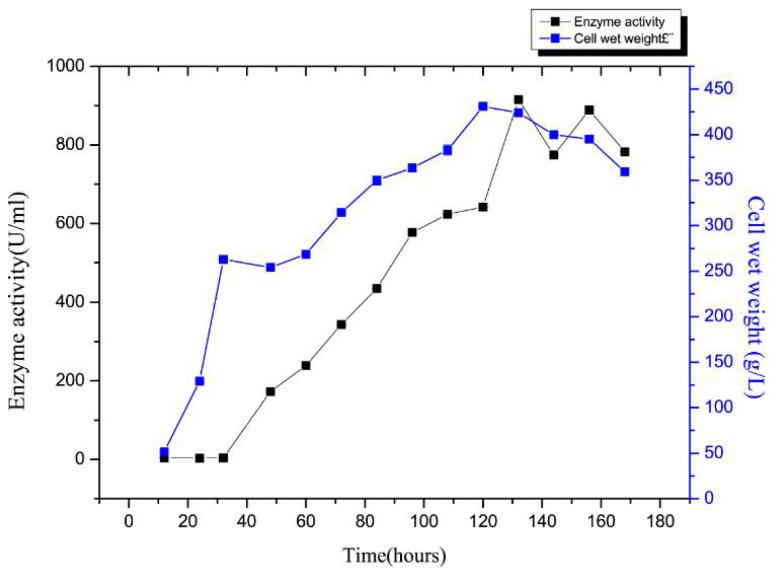
Enzyme activity and cell growth variation curves during rSAGL expression in fermentor. Samples were collected at 12, 24, 32, 48, 60, 72, 84, 96, 108, 120, 132, 144, 156, and 168 h. All experiments were conducted in triplicate, and the data were expressed as mean ± standard deviation.

Next, enzyme activity and cell growth variation curves during rSAGL expression were determined. The results were shown in Figure 9. After the glycerol batch phase, glycerol fed-batch phase, and methanol fed-batch phase, the wet cell weight reached 424 g/L, the maximum alginate lyase activity in the supernatant of BSM medium reached 915.5 U/mL (Figure 9). By referring to the specific activity of 4044 U/mg protein, we concluded that the yield of recombinant alginate lyase enzyme reached 226.4 μg/mL. This yield of 915.5 U/mL was 9.8 times higher than that of the shake flask fermentation, which was 95 U/mL (equal to 23.1 μg/mL) (Figure 1a). The yield of rSAGL produced by *P. pastoris* was impressively higher than yields of other recombinant alginate lyases produced in *E. coli* system in previous reports (Table 3). The highest yield of them was 496 U/mL (800 mg/L) [5]. In only one example of recombinant alginate lyase produced in *P. pastoris* system, the yield data was not shown [4]. Our study was the first report of high-level recombinant expression of alginate lyase in the *P. pastoris* system (Table 3). It was speculated that *P. pastoris* was a more suitable system than *E. coli* for high-level production of recombinant alginate lyase.

### 2.9. Production and Analysis of Brown Alginate Oligosaccharide

**Figure 10:**
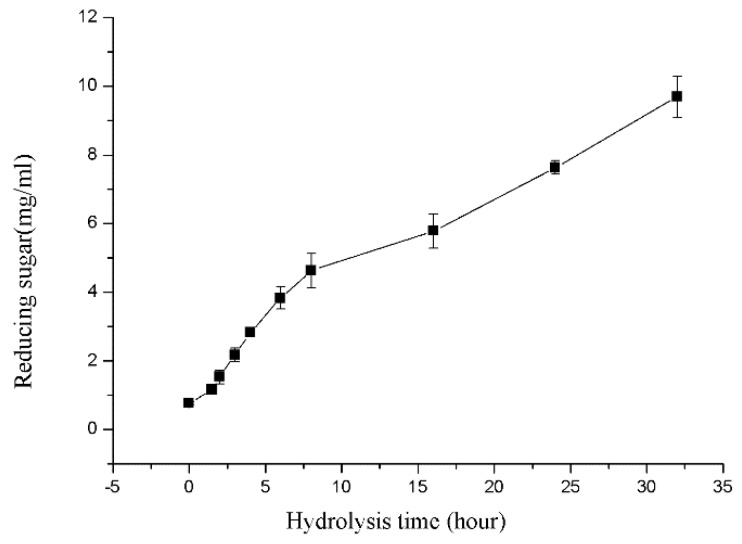
Reducing sugar concentration variation curves during large scale hydrolysis of sodium alginate substrate. Samples were collected at 1.5, 2, 3, 4, 6, 8, 16, 24, and 32 h. All experiments were conducted in triplicate, and the data were expressed as mean ± standard deviation.

Sodium alginate was used as substrate to produce brown alginate oligosaccharide in large scale. For convenience, crude enzyme of rSAGL, which was supernatant of yeast fermentation culture, was directly used in production. In the transformation course, the amount of released reducing sugar that was calculated by using glucose equivalent in the reaction mixture was shown in Figure 10.

**Figure 11:**
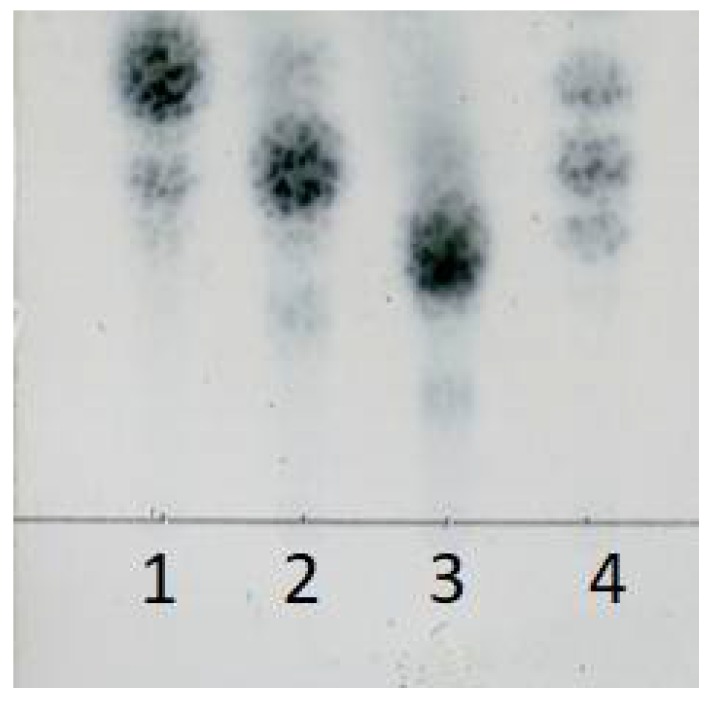
TLC analysis of oligosaccharide product of large-scale hydrolysis of sodium alginate substrate. Lane 1: l-diguluronic acid disodium salt and l-dimannuronic acid disodium salt; lane 2: l-triguluronic acid disodium salt and l-trimannuronic acid disodium salt; lane 3: l-tetraguluronic acid disodium salt and l-tetramannuronic acid disodium salt; lane 4: oligosaccharide product solution. Above standard compounds were used by equal molar mass in test.

The final concentration of reducing sugar in the mixture reached 9.51 g/L at the end of 32 h hydrolysis course. After filtration, the oligosaccharide solution was freeze-dried to solid powder. Finally, 97.2% of transformation rate of sodium alginate was obtained. TLC (Thin layer chromatography) results showed that main oligosaccharide products were disaccharide, trisaccharide, and tetrasaccharide (Figure 11). These results suggested that rSAGL with high activity and thermal stability was a good candidate enzyme for industrial production of brown alginate oligosaccharide with low molecular weight.

## 3. Materials and Methods

### 3.1. Expression of Alginate Lyase in E. coli System

For *E. coli* expression, pET28a vector and host cell BL21(DE3) were purchased from Novagen (Merck, Darmstadt, Germany). The 923 base pairs (bp) nucleotide acid sequence of an alginate lyase encoding gene from *Flavobacterium* sp. H63 was named as *nagl* and registered in Genbank with accession number MH234061. This *nagl* gene was ligated into pET28a vector at NdeI and HindIII sites. This recombinant vector named pET28a-nagl was confirmed by sequencing and transformed into BL21(DE3) competent cells. The induction, expression, and purification of recombinant alginate lyase of *nagl* was proceeded according to protocols of Molecular Cloning Handbook: A Laboratory Manual (Third Edition). 

### 3.2. Strain and Plasmid of Pichia System

Both the yeast expression vector pPICZαA and yeast host strain *Pichia pastoris* X33 were purchased from Invitrogen (Thermo Fisher, Waltham, MA, USA).

### 3.3. Gene Cloning and Construction of Recombinant Yeast Expression Vector In Vitro

The alginate lyase encoding gene from *Flavobacterium* sp. H63 was optimized according to *Pichia pastoris* codon usage bias. This gene sequence was named as *sagl* and registered in Genbank with accession number MG792316. The recombinant expression vector was constructed by inserting this *sagl* gene into pPICZαA vector at EcoRI and NotI sites. This recombinant vector was confirmed by sequencing.

### 3.4. Transformation and Colony Screening of P. pastoris

The transformation and expression protocols of *P. pastoris* were mainly according to the *P. pastoris* expression manual of previously Invitrogen; Life Technologies (Carlsbad, CA, USA). Briefly, the recombinant pPICZαA vector harbouring alginate lyase gene was linearized by using SacII and transformed into *P. pastoris* X33 competent cell. All positive recombinant yeast colonies were inoculated on BMGY plates. Two days later, all colonies were transferred onto BMMY plates supplemented with 10 g/L sodium alginate (CAS 9005-38-3, sigma 180947) and 10 g/L methanol. Five days later, each plate was sprayed with 1 mL of 10% (*w*/*v*) hexadecylpyridinium chloride (CAS 6004-24-6, sigma c9002) solution. The diameters of both yeast colonies and hydrolytic ring around the colonies were measured. The yeast colony with high ratio of the diameter of hydrolytic ring to that of yeast colony was selected for further shaking flask fermentation test.

### 3.5. Shaking Flask Fermentation Test

The yeast fermentation in 250 mL shaking flask was carried out according to the *P. pastoris* expression manual. A recombinant yeast colony was inoculated in 100 mL of BMGY medium containing 100 mM potassium phosphate of pH 6.0, 1.34% (*w*/*v*) yeast nitrogen base (YNB), 4 × 10^−5^% (*w*/*v*) biotin, and 10 g/L glycerol. After culture at 28 °C for 48 h, the cells were collected by centrifugation and re-suspended in 25 mL of BMMY medium supplemented with 100 mM potassium phosphate of pH 6.0, 1.34% (*w*/*v*) of YNB and 4 × 10^−5^% (*w*/*v*) biotin. The initial optical density value of culture at 600 nm was adjusted to 1.0. The incubation temperature was set at 28 °C, the rotate speed was 250 rpm. The methanol with working concentration of 5 g/L was feed at 0, 24, 48, 72, 96, 120, and 144 h. During the induction course, supernatants samples were collected at 0, 24, 48, 72, 96, 120, 144, and 168 h. Protein expression in samples was analyzed by using SDS-PAGE.

### 3.6. Recombinant Enzyme Purification, Western Blotting and Activity Assay

The recombinant SAGL enzyme was purified from shaking flask medium supernatant using Ni-NTA resin. The substrate for activity assay was 10 mg/mL of sodium alginate solution prepared using 100 mM Na_2_HPO_4_-NaH_2_PO_4_ buffer of pH 6.0. The reaction mixture composed of 1 mL of substrate and 5 μL of enzyme was incubated at 50 °C for 10 min without stirring. The reducing sugar content was determined by using DNS reagent. Glucose was used in standard curve construction. One enzyme unit was defined as the amount of enzyme that could release one micro molar glucose per minutes in above reaction. The protein concentration of the enzyme was assayed using the Bradford method. The deglycosylation treatment was done with EndoH (NEB). A total of 20 μL of purified rSAGL enzyme was incubated with 2.0 IU of EndoH at 37 °C for 3 h according to the instructions of manufacturer. Then, the reaction mixture was analyzed using SDS-PAGE. The purified rSAGL was also tested using western blotting. The first antibody was mouse anti His antibody, and the second antibody was HRP-labeled Goat Anti-Mouse IgG (H + L). DAB Horseradish Peroxidase Color Development Kit was used to stain target protein band.

### 3.7. Substrate Specificity Determination

PolyM (poly-β-d-mannuronate), polyG (poly-α-l-guluronate), and sodium alginate were dissolved respectively in 100 mM Na_2_HPO_4_-NaH_2_PO_4_ buffer (pH 7.0) with 4 mg/mL working concentration and used as substrate in this test. PolyM and polyG were purchased from BZ OLIGO Company (Qingdao, China). Reactions were initiated by adding appropriate enzyme, and the amount of yielded unsaturated uronic acid was monitored by recording the absorbance of the reaction mixture at 235 nm.

### 3.8. Characterization of Optimal Reaction Temperature and pH

The optimal catalytic temperature was investigated in the range from 40 °C to 60 °C. The reaction buffer was 100 mM Na_2_HPO_4_-NaH_2_PO_4_ buffer of pH 7.0. The optimal catalytic pH value was investigated in the range from 3.0 to 11.0. The reaction buffers were 50 mM NaAc-HAc buffer (pH 3.0, 4.0, 5.0, 6.0); 50 mM Na_2_HPO_4_-NaH_2_PO_4_ buffer (pH 6.0, 7.0, 8.0); and 50 mM glycine-NaOH buffer (pH 8.0, 9.0, 10.0, 11.0). The reaction mixture composed of 10 mg sodium alginate and 5 μL enzyme preparation in 1 mL buffer was incubated at 50 °C for 10 min without stirring. The reducing sugar content was determined using DNS reagent.

### 3.9. Characterization of Thermal Stability

The test of T50 value which was important parameters of enzyme thermal stability was proceeded as in previous reports [6]. The preparation of enzyme was incubated in 50 mM Na_2_HPO_4_-NaH_2_PO_4_ buffer (pH 7.0) at 25–55 °C for 30 min. After incubation, these samples were transferred into an ice-bath for quick cooling. The residual enzyme activity of sample was determined using the standard method. The plot of T50 was made using temperature as horizontal coordinates and ratio of residual activity and initial activity as vertical coordinates. The test was conducted three times for every sample.

Next, the buffer effect on thermal stability was tested. Six types of buffer including 50 mM Na_2_HPO_4_-NaH_2_PO_4_ buffer (pH 6.0, 7.0, 8.0), 50 mM Tris-HCl buffer (pH 7.0, 8.0), and 50 mM NaAc-HAc buffer (pH 6.0) were used in tests. The incubation temperature for the buffer test was adjusted to 50 °C. The residual activities of enzyme samples during incubation were determined at 30 min intervals.

### 3.10. Characterization of Metal Ions Effects

Metal ions effects were tested at 1 mM and 10 mM working concentration. Na^+^ and K^+^ with concentration range from 10 mM to 500 mM were tested separately. The reaction condition was same to the above activity assay condition.

### 3.11. ESI-MS and TLC Analysis of End Oligosaccharide Product

The oligosaccharide product prepared through hydrolysis of sodium alginate substrate was analyzed by TLC and ESI-MS. In the hydrolysis reaction mixture, 0.1 g of sodium alginate and 9 mL of 100 mM NaH_2_PO_4_-Na_2_HPO_4_ buffer (pH 6.0) were mixed in the beaker by vigorous stirring for 30 min. After adding 10 units of purified enzyme, the reaction mixture was incubated in a water-bath of 50 °C. The stirring speed was adjusted to 200 rpm. After reaction course of 8 h, protein and salts in the hydrolyzes were removed using Savage reagent and nanofiltration membrane (ESNA1 4040, Nitto Group Company, Oceanside, CA, USA), separately. The Savage reagent was a mixture of chloroform/1-butanol with volume ratio of 4:1. The oligosaccharide product was analyzed by using ESI-MS. The hydrolyzed products were concentrated, freeze-dried, and redissolved in 1 mL methanol. An amount of 2 μL of supernatant was loop-injected to Waters 2795 HPLC system with dual wavelength UV detector, and ZQ single quadrupole MS with electrospray ionization source. The oligosaccharides products were detected in a positive-ion mode using the following settings: ion source voltage, 4.5 kV; capillary temperature, 275–300 °C; Tube lens, 250 V; sheath gas, 30 arbitrary units (AU); scanning the mass range, 150–2000 *m*/*z*. In the TLC analysis of oligosaccharide product, TLC plate (Silica Gel 60 F 254, Merck) was used. The solvent system was 1-butanol/formic acid/water (4:6:1, *v*/*v*). After chromatography plates was sprayed with 10% (*v*/*v*) sulfuric acid in ethanol and heated at 130 °C for 5 min to visualize spots of oligosaccharide. Six standard oligosaccharide compounds of l-diguluronic acid salt, l-triguluronic acid salt, l-tetraguluronic acid salt, l-dimannuronic acid salt, l-trimannuronic acid salt, and l-tetramannuronic acid salt were purchased from BZ OLIGO Company (Qingdao, China). The above six compounds all are disodium salts. They were mixed and used as markers in chromatography.

### 3.12. High Cell-Density Fermentation of Recombinant Alginate Lyase in P. pastoris

In the course of high cell-density fermentation, basal salt medium (BSM) was used. The medium preparation protocol was referred to “Pichia Expression Manual” and “Pichia Fermentation Process Guidelines” of Invitrogen. Recombinant *P. pastoris* strain was inoculated into a flask containing 300 mL BMGY medium, and incubated at 30 °C, 250 rpm for 48 h, and then 300 mL BMGY medium were transferred to 6 L BMGY medium in a 10-L Biostat B plus fermenter (B. Braun Biotech International, GmbH, Melsungen, Germany). The system was maintained at 30 °C, pH 5.3 (with 28% NH_4_OH) and 30% dissolved O_2_. After the glycerol was exhausted, 12 mL PTM1 trace salts/L (Invitrogen) with 50% (*v*/*v*) glycerol was fed continuously. The glycerol feeding rate was 18 mL/h/L for another 2–4 h until the OD 600 nm of the cell culture reached 180. Next, 100% methanol with 12 mL PTM1 trace salts/L was added to induce the expression of alginate lyase. The initial methanol concentration was adjusted to 30 g/L. The ratio of methanol content to wet weight of cells was about 0.2. This ratio was maintained by methanol feed-batch. About 10 mL cell culture was collected every 24 h for SDS-PAGE, western blotting, and enzyme activity assays. In western blotting, the first antibody was mouse anti His antibody and the second antibody was HRP-labeled Goat Anti-Mouse IgG (H + L). DAB Horseradish Peroxidase Color Development Kit was used to stain target protein band. The fermentation course lasted 168 h. The supernatant of culture separated by centrifugation was directly used as crude rSAGL enzyme.

### 3.13. Enzymatic Production of Alginate Oligosaccharide

Sodium alginate was used as substrate to produce oligosaccharide. Amounts of 100 kg substrate and 900 L NaH_2_PO_4_-Na_2_HPO_4_ buffer (100 mM, pH 6.0) were mixed in a stainless reactor by vigorous stirring for 30 min. An amount of 300 mL of crude rSAGL enzyme with about 300 K enzyme units were added into the above mixture. The reaction temperature and the stirring speed were adjusted to 50 °C and 200 rpm. The reducing sugar contents of mixtures in reaction course were determined at intervals by using the DNS method. After 32 h of transformation, the temperature of mixture rose to 70 °C for 15 min to inactive enzyme and stop reaction. After centrifugation, the supernatant was harvested for further filtration using 0.22 μm microporous filters. Next, the filtrate solution containing oligosaccharide was freeze-dried until the total solid reached constant weight. Oligosaccharide product weight was obtained by subtracting the weight of NaH_2_PO_4_-Na_2_HPO_4_ in the buffer from the solid weight. The transformation rate of substrate was the percentage of oligosaccharide weight to initial weight of sodium alginate. Freeze-dried oligosaccharide powder was redissolved by water and analyzed by using TLC.

## 4. Conclusions

In conclusion, a recombinant yeast strains high-level-expressed alginate lyase was successfully constructed in this study. This recombinant alginate lyase with high thermal stability and specific activity was high-level produced by high density fermentation. The yield of 226.4 μg/mL (915.5 U/mL) was the highest record of alginate lyase production so far, and the recombinant enzyme was successfully applied in production of brown alginate oligosaccharide. It was speculated that the *Pichia pastoris* system was more suitable than the *E. coli* system for recombinant expression of marine polysaccharide transformation enzyme. Further investigation of this enzyme about molecular modification has been proceeded.

## Figures and Tables

**Table 1:** Metal ions effect on the activity of rSAGL.

Metal Ion	Ion Concentration	Relative Activity
Mn^2+^	1 mM	0
	10 mM	0
Cu^2+^	1 mM	0
	10 mM	0
Co^2+^	1 mM	0
	10 mM	0
Ca^2+^	1 mM	74 ± 0.2
	10 mM	0
Mg^2+^	1 mM	106 ± 0.6
	10 mM	93 ± 1.3
	100 mM	147 ± 0.9
Ag^+^	1 mM	0
	10 mM	0
Zn^2+^	1 mM	15 ± 0.4
	10 mM	0
Ni^+^	1 mM	29 ± 0.7
	10 mM	0
Fe^3+^	1 mM	5.3 ± 0.2
	10 mM	14 ± 1.1
Control enzyme	0	100 ± 3.4

**Table 2:** Na and K ions effect on activity.

Ions Concentration (mM)	Relative Activity (%)	
	NaCl	KCl
0	100	100
10	201 ± 2.4	237 ± 5.6
20	292 ± 4.1	274 ± 3.5
50	168 ± 5.4	270 ± 1.7
80	135 ± 8.3	344.4 ± 2.9
100	246 ± 1.2	325 ± 2.7
150	120 ± 5.0	300 ± 6.2
200	131.5 ± 6.1	283.7 ± 7.5
300	140 ± 3.3	215 ± 1.8
400	215 ± 4.6	147 ± 0.4
500	197 ± 5.3	177 ± 8.0

**Table 3:** Catalytic property comparisons of some endo-type alginate lyases.

Origin	Source	Substrate Preference	Optimum Catalytic Temperature and Thermal Stability	Specific Activity	Main End Product	Yield	Reference
*Microbulbifer* sp. ALW1	*E. coli*	Poly G and alginate	45 °C and retained 68% activity at 45 °C for 1 h	1.49 U/mg	DP 2,3	N.D.	[1]
*Flavobacterium* sp. UMI-01	*E. coli*	Poly M	55 °C and inactivated at 50 °C for 30 min	23478 EU/mg	DP 2,3,4	168 EU/mL	[2]
*Pseudomonas aeruginosa* PAOI	*P. pastoris*	Poly (MG)	40 °C and inactivated at 50 °C for 30 min	2440 U/mg.	DP 2,3	21 U/mL	[4]
*Flammeovirga* sp. MY04:	*E. coli*	Poly G	40 °C and retained 80% of activity at 40 °C for 2 h	620 U/mg	DP 2,3,4	496 U/mL (800 mg/L)	[5]
*Nitratiruptor* sp. SB155-2	*E. coli*	Poly M	70 °C and retain 20% of activity by incubation at 50 °C for 16 h	1620 U/mg.	DP 3,4,5,6	1.944 U/mL (1.2 mg/L)	[6]
*Bacillus* sp. Alg07	native	Poly M	40 °C and retained 50% activity at 50 °C for 0.75 h	8306.7 U/mg	DP 2,3,4	N.D.	[7]
*Vibrio* sp. NJU-03	*E. coli*	Poly G	30 °C and retained 40% activity at 40 °C for 30 min	6468.99 U/mg	DP 2,3,4	N.D.	[8]
*Pseudoalteromonas* sp. SM0524	*E. coli*	Poly M	30 °C and lost 80% activity at 40 °C for 15 min	62.6 U/mg	DP 2,3,4	N.D.	[9]
*Vibrio* sp. SY08	native	Poly G and Poly M	40 °C and retained 75% activity at 40 °C for 2 h	1070.2 U/mg	DP 2	2.247 U/mL (2.1 mg/L)	[10]
*Vibrio* sp. QY105	native	Poly G and alginate	38 °C and retained 58% of activity at 50 °C for 20 min	2152 U/mg	DP 2,3,4,5	15.8 U/mL	[11]
*Pseudomonas* sp. E03	*E. coli*	Poly M	30 °C and lost 50% activity at 50 °C for 30 min	222 EU/mg	DP 2,3,4,5	N.D.	[12]
*Sphingomonas* sp. A1-II	*E. coli*	Poly G	70 °C and lost 50% of activity at 50 °C for 10 min	109 U/mg	DP 3,4	3040 U/L	[13]
*Saccharophagus degradans* 2–40	*E. coli*	Poly G and Poly M	50 °C and lost 58% of activity at 50 °C for 30 min	4.6 U/mg	DP 2,3,4,5	41.4 U/L	[14]
*Flavobacterium* S20	*E. coli*	Poly G	45 °C and lost 20% of activity at 45 °C for 60 min	365.38 U/mg	DP 5,6,7	19.6 U/mL	[15]
*Stenotrophomas maltophilia* KJ-2	*E. coli*	Poly (MG)	40 °C and inactivitaed at higher than 40 °C for 30 min	848.3 U/mg	DP 2,3,4	N.D.	[16]
*Flavobacterium* sp. H63	*P. pastoris*	Poly M and alginate	45 °C and retained 49.0% activity at 50 °C for 72 h	4044 U/mg	DP 2,3,4	915.5 U/mL (226.4 μg/mL)	This study

## References

[1] Zhu Y., Wu L., Chen Y., Ni H., Xiao A., Cai H. (2016). Characterization of an extracellular biofunctional alginate lyase from marine *Microbulbifer* sp. ALW1 and antioxidant activity of enzymatic hydrolysates. Microbiol. Res..

[2] Inoue A., Takadono K., Nishiyama R., Tajima K., Kobayashi T., Ojima T. (2014). Characterization of an Alginate Lyase, FlAlyA, from *Flavobacterium* sp. Strain UMI-01 and Its Expression in *Escherichia coli*. Mar. Drugs.

[3] Kang L.X., Chen X.M., Fu L., Ma L.X. (2012). Recombinant expression of chitosanase from *Bacillus subtilis* HD145 in *Pichia pastoris*. Carbohyd. Res..

[4] Yue M.M., Gong W.W., Qiao Y., Ding H. (2016). A Method for Efficient Expression of *Pseudomonas aeruginosa* Alginate Lyase in *Pichia pastoris*. Prep. Biochem. Biotechnol..

[5] Han W., Gu J., Cheng Y., Liu H., Li Y., Li F. (2015). Novel Alginate Lyase (Aly5) from a Polysaccharide-Degrading Marine Bacterium, *Flammeovirga* sp. Strain MY04: Effects of Module Truncation on Biochemical. Characteristics, Alginate Degradation Patterns, and Oligosaccharide-Yielding Properties. Appl. Environ. Microb..

[6] Inoue A., Anraku M., Nakagawa S., Ojima T. (2016). Discovery of a Novel Alginate Lyase from *Nitratiruptor* sp. *SB*155-2 Thriving at Deep-sea Hydrothermal Vents and Identification of the Residues Responsible for Its Heat Stability. J. Biol. Chem..

[7] Chen P., Zhu Y.M., Men Y., Zeng Y., Sun Y.X. (2018). Purification and Characterization of a Novel Alginate Lyase from the Marine Bacterium *Bacillus* sp. Alg07. Mar. Drugs.

[8] Zhu B.W., Sun Y., Ni F., Ning L.M., Yao Z. (2018). Characterization of a new endo-type alginate lyase from *Vibrio* sp. NJU-03. Int. J. Biol. Macromol..

[9] Chen X.L., Sheng D., Fei X., Fang D., Li P.Y., Zhang X.Y., Zhou B.C., Zhang Y.Z., Xie B.B. (2016). Characterization of a New Cold-Adapted and Salt-Activated Polysaccharide Lyase Family 7 Alginate Lyase from *Pseudoalteromonas* sp. SM0524. Front. Microbiol..

[10] Li S., Wang L., Hao J., Xing M., Sun J., Sun M. (2017). Purification and Characterization of a New Alginate Lyase from Marine Bacterium *Vibrio* sp. SY08. Mar. Drugs.

[11] Wang Y., Guo E.W., Yu W.G., Han F. (2013). Purification and characterization of a new alginate lyase from a marine bacterium *Vibrio* sp.. Biotechnol. Lett..

[12] Zhu B.W., Huang L.S., Tan H.D., Qin Y.Q., Du Y.G., Yin H. (2015). Characterization of a new endo-type polyM-specific alginate lyase from *Pseudomonas* sp.. Biotechnol. Lett..

[13] Hye-Jin Y., Hashimoto W., Osamu Miyake M.O., Mikami B., Murata K. (2000). Overexpression in *Escherichia coli*, purification, and characterization of *Sphingomonas* sp. A1 alginate lyases. Protein Expr. Purif..

[14] Kim H.T., Ko H.J., Kim N., Kim D., Lee D., Choi I.G., Woo H.C., Kim M.D., Kim K.H. (2012). Characterization of a recombinant endo-type alginate lyase (Alg7D) from *Saccharophagus degradans*. Biotechnol. Lett..

[15] Huang L., Zhou J., Li X., Peng Q., Lu H., Du Y. (2013). Characterization of a new alginate lyase from newly isolated *Flavobacterium* sp. S20. J. Ind. Microbiol. Biotechnol..

[16] Lee S.I., Choi S.H., Lee E.Y., Kim H.S. (2012). Molecular cloning, purification, and characterization of a novel polyMG-specific alginate lyase responsible for alginate MG block degradation in *Stenotrophomas maltophilia* KJ-2. Appl. Microbiol. Biotechnol..

